# Editorial: New insights into synaptic plasticity in fear conditioning

**DOI:** 10.3389/fnsyn.2023.1270701

**Published:** 2023-09-13

**Authors:** Ana P. Crestani, Ana Cicvaric, Adelaide P. Yiu

**Affiliations:** ^1^Center for Neuroscience, University of California, Davis, Davis, CA, United States; ^2^Dominick P. Purpura Department of Neuroscience, Albert Einstein College of Medicine, New York, NY, United States; ^3^Independent Researcher, Toronto, ON, Canada

**Keywords:** fear conditioning, memory extinction, hippocampal formation/hippocampus, amygdala, ventral tegmental area (VTA)

The fear conditioning task in rodents serves as a valuable tool for studying learning and memory, as well as enhancing our understanding of post-traumatic stress disorder (PTSD) and other stress-related disorders. By utilizing controlled experimental paradigms, traumatic experiences can be mimicked in rodents, allowing investigation of underlying neurobiological mechanisms. During the fear conditioning task, animals learn to associate a neutral stimulus, such as a tone or a context, with an aversive, unconditioned stimulus, most often a mild foot shock. Then, the previously neutral stimulus becomes conditioned stimulus, and when the animals are re-exposed to it, they show fear response. The fear response is most commonly measured as the percentage of time spent freezing, which is defined as the time the animal spends motionless, except for cardiac and respiratory movement.

While several brain regions are involved in processing and storing of these associative episodic-like memories, the spatial attributes rely primarily on the hippocampus, while emotional valence depends mostly on the amygdala (Maren, 2001, 2008; Josselyn, 2010; Izquierdo et al., 2016). Understanding how these two hub regions are regulated, and function in coordination with other brain regions, and how stress hormones and synaptic mechanisms influence brain circuits involved in fear and emotional processing, may potentially bring new insights for treating maladaptive memories. The present Research Topic encompasses diverse studies examining brain regions recruited for fear and emotional processing on molecular, circuit and behavioral level.

Hippocampal formation and entorhinal cortex show a high degree of interconnectivity and functional interaction between the hippocampus and MEC is crucial for spatial memory processing (Hafting et al., 2005; Sanders et al., 2015). Hartner and Schrader's article explores the effects of stress hormones, norepinephrine (NE), and glucocorticoids on inhibitory signaling in the Layer 2 of medial entorhinal cortex (MEC_LII) circuitry. The authors show the ability of NE to increase the frequency and amplitude of inhibitory inputs to the MEC, an effect that is cell-specific, mediated by the activation α1 adrenergic receptors, and modulated by co-administration of corticosteroids. This study sheds light on how stress influences underlying mechanisms of fear memory formation by demonstrating the ability of stress hormones to alter inhibitory synaptic inputs within the MEC-LII.

Asede et al. focus on the role of ErbB4, a receptor for neuregulin that is exclusively expressed in GABAergic neurons of the central nervous system, in intercalated cell clusters (ITCs) of the amygdala. As the amygdala plays a crucial role in fear conditioning (LeDoux, 2000, 2007; Krabbe et al., 2018), understanding the synaptic mechanisms involved in its circuitry is essential. This study focused on the medial paracapsular ITC (mpITC) and its thalamic inputs. The authors provide insights into how the deletion of ErbB4 differentially affects inhibitory and excitatory circuits, thus disturbing the excitation-inhibition balance in the amygdala and how lack of ErbB4 compromises LTP of the thalamo-mpITC synapses, contributing to further understanding of the functional role ErbB4 has during fear conditioning.

Ferrara et al. scrutinize the two procedurally similar but distinct processes: memory extinction and memory interference during reconsolidation following fear conditioning. Extinction involves reducing fear responses through numerous repeated exposures to either conditioned stimulus (CS) or conditioning context on its own, without the previously predictive unconditioned stimulus, such as mild foot shock. This leads to the formation of a new, inhibitory memory (Quirk and Mueller, 2008; Pape and Pare, 2010). In contrast, interference during reconsolidation requires only a few CS presentations and modifies the original fear memory (Nader and Hardt, 2009; Lee et al., 2017). This review examines the behavioral and neurobiological mechanisms underlying these two processes, providing valuable insights into the boundary conditions of post-conditioning cue exposure.

Liu et al. investigate the neural circuitry involved in assigning emotional valence to stimuli. The central amygdala (CeA) and the ventral tegmental area (VTA) are key regions involved in emotional processing and assignment of valence (Namburi et al., 2016; Tye, 2018). The authors for the first time identified neural populations that project to both the CeA and the VTA in the posterior bed nucleus of the stria terminalis (pBNST), pedunculopontine tegmental nucleus (PPTg), and the anterior part of the basomedial amygdala (BMA). The study shows that the positive valence is induced by activating pBNST neurons, while PPTg activation produces a negative valence, and valence processing is not influenced by BMA activation. These findings contribute to our understanding of the neural mechanisms underlying emotional regulation and point to possible important targets for mood disorder treatment.

The aim of this Research Topic is to offer New Insights into Synaptic Plasticity in Fear Conditioning. While previous studies on fear conditioning have mostly focused on examining the triad of brain regions crucial for fear conditioning -hippocampus, amygdala, and medial prefrontal cortex-, the articles in this Research Topic broaden the scope by investigating circuits and molecular mechanisms of other brain regions that play important role in fear conditioning and emotion processing. Additionally, this Research Topic is putting a spotlight on the inhibitory transmission, and the inhibition/excitation balance. The inhibitory transmission has been historically understudied. Albeit interneurons are fewer in number in areas crucial for fear conditioning they are not passive gain regulators but exert their robust inhibition via intricate network of connections on the excitatory projection neurons, thus shaping the network activity (Lucas and Clem, 2018). Hence, with this Research Topic we hope to highlight the complex interplay between brain circuits involved in fear and emotional processing and provide a better understanding of the neurobiological mechanisms that potentially support stress-related disorders.

## Author contributions

ACr: Writing—original draft. ACi: Writing—review and editing. AY: Writing—original draft.
